# Supplementary material for: Kramers nodal line metals
Source: Nat Commun. 2021 May 24;12:3064. doi: 10.1038/s41467-021-22903-9 (PMC8144424; doi:10.1038/s41467-021-22903-9)
Supplement: Supplementary file 1 — Supplementary Information [file 41467_2021_22903_MOESM1_ESM.pdf]

Supplementary Information – Kramers Nodal Line Metals

Ying-Ming Xie[1], Xue-Jian Gao[1], Xiao Yan Xu[2], Cheng-Ping Zhang[1], Jin-Xin Hu[1], Jason Z. Gao[1], and K. T. Law[1*]
[1] *Department of Physics, Hong Kong University of Science and Technology, Clear Water Bay, Hong Kong, China and*
[2] *Department of Physics, University of California at San Diego, La Jolla, California 92093, USA*

Supplementary Note 1. DFT CALCULATIONS

Throughout this work, the Vienna Ab initio Simulation Package (VASP) [1] with the projector-augmented wave method [2] and the Perdew-Berke-Ernzerhof's (PBE) exchange-correlation functional in the generalized-gradient approximation [3, 4] was used to perform the first-principles density functional theory (DFT) calculations [5]. Information about calculated materials such as the lattice structures was obtained mainly from several material databases, *e.g.* the Materials Project [6], the Topological Materials Database [7], the Inorganic Crystal Structure Database (ICSD) [8] and the TopoMat Database [9].

To further look into the topological properties as well as to plot the special spindle torus and octdong Fermi surfaces of KNLMs, maximally localized generalized Wannier bands of some KNLMs (such as BiTeI, HgSe, $Bi_2Te_2Br_2O_9$ and Cr_2AgBiO_8) were projected from the first-principles results through the Wannier90 package [10, 11] linked to VASP.

The open-source package WannierTools was used for post-processing of the Wannier tight-binding Hamiltonian [12]. These processes include the Fermi surface plotting, the calculation of Fermi arcs, surface states and chiral charges of Kramers Weyl points.

Supplementary Note 2. A GENERAL PROOF OF THE EXISTENCE OF KNLS IN ACHIRAL CRYSTALS

A. Notations

The space group G consists of all operations $\{R_\alpha|\mathbf{t}\}$ which leave a given lattice invariant, where the space group operator $\{R_\alpha|\mathbf{t}\}\mathbf{r} = \mathbf{r}' = R_\alpha\mathbf{r}+\mathbf{t}$. A space group is called symmorphic if there is a point such that all symmetries are the product of a symmetry fixing this point and a translation. For symmorphic space groups, the point group $\mathcal{G}$ is isomorphic to the factor group G/T with T as the translational group forming by translational operations $\{E|\mathbf{t}\}$ that leave the lattice to be invariant, E is identity operation. On the contrary, nonsymmorphic space groups cannot be represented as semi-direct product groups of a discrete translation group T and a corresponding point group $\mathcal{G}$.

The little group $G_\mathbf{k}$ of a wave vector $\mathbf{k}$ is formed by the set of space group operations $\{R_\alpha|\mathbf{t}\}$ such that $R_\alpha\mathbf{k} = \mathbf{k} + \mathbf{G}_i$, $\mathbf{G}_i$ is the reciprocal lattice vector. For our purpose, it is sufficient to determine the reps of Herring's little group ${}^HG^\mathbf{k} = G^\mathbf{k}/T^\mathbf{k}$, where $T^\mathbf{k}$ is the group of the translational symmetry operations $\{E|\mathbf{t}\}$ with $\exp(-i\mathbf{k}\cdot\mathbf{t}) = 1$. The ${}^HG^\mathbf{k}$ in general can be identified with one of the abstract groups (AGs) given in [13]. For a symmorphic space group, the Herring's little group ${}^HG^\mathbf{k}$ is always isomorphic to a point group $\mathcal{G}_\mathbf{k}$. Throughout this work, the little group refers to the Herring's little group, where the integer translations have been factored away.

When there exhibits an additional anti-unitary symmetry $\mathcal{T}$ with $\mathcal{T}^2 = -1$, such as time-reversal symmetry, that leaves $\mathbf{k}$ to be invariant, the symmetry group becomes $G_\mathbf{k} + \mathcal{T}G_\mathbf{k}$. In this case, the states at TRIMs are described by the corepresentations of the symmetry group $G_\mathbf{k} + \mathcal{T}G_\mathbf{k}$. And since the translational operations is not essential here, we can directly use the corepresentations of the Herring's little group ${}^HG^\mathbf{k}$ to label the states at TRIMs. Furthermore, according to the theory of corepresentations [13], if a corepresentation D^{Γ_i} is real or complex, the corep is irreducible and the degeneracies at TRIMs will be doubled due to the anti-unitary symmetry, while if D^{Γ_i} is pseudo-real, the corepresentation becomes reducible and there is no extra degeneracy from this anti-unitary symmetry. A more systematic introduction to corepresentations can be found in Ref. [13].

B. Symmetry properties of the SOC term

The bands near a TRIM $\mathbf{k}_0$ with a two-fold degeneracy can be described by a two-band Hamiltonian

$$H(\mathbf{k}) = f_0(\mathbf{k})\sigma_0 + \boldsymbol{f}(\mathbf{k})\cdot\sigma, \quad (1)$$

where $\boldsymbol{f}(\mathbf{k})\cdot\sigma$ denotes the spin-orbit coupling term (SOC), and σ are Pauli matrices operating on spin space $|\pm\frac{1}{2}\rangle$. This Hamiltonian needs to respect the symmetry $\mathcal{T}\times\mathcal{G}_{\mathbf{k}_0}$, where $\mathcal{T} = i\sigma_y K$ with K as complex conjugate is time-reversal symmetry, and $\mathcal{G}_{\mathbf{k}_0}$ is the point group symmetry that the Herring's little group ${}^HG^\mathbf{k}$ is isomorphic to. The time-reversal symmetry requires $\boldsymbol{f}(\mathbf{k}) = -\boldsymbol{f}(-\mathbf{k})$, while the constraint imposed by a symmetry operation R in $\mathcal{G}_{\mathbf{k}_0}$ is $H(\mathbf{k}) = U^{-1}_{1/2}(R)H(R\mathbf{k})U_{1/2}(R)$,

* phlaw@ust.hk

i.e.,

$$\begin{aligned}\boldsymbol{f}(\mathbf{k})\cdot\sigma &= U_{1/2}^{-1}(R)\boldsymbol{f}(R\mathbf{k})\cdot\sigma U_{1/2}(R)\\ &= \mathrm{Det}(R)\boldsymbol{f}(R\mathbf{k})\cdot(R\sigma)\\ &= \mathrm{Det}(R)R^{-1}\boldsymbol{f}(R\mathbf{k})\cdot\sigma. \end{aligned}\tag{2}$$

$$\boldsymbol{f}(\mathbf{k}) = \mathrm{Det}(R)R^{-1}\boldsymbol{f}(R\mathbf{k}),\tag{3}$$

where $R\in O(3)$, and $U_{1/2}(R)$ is the $SU(2)$ representation of R.

C. Symmetry transformation properties of the linear term

When $\boldsymbol{f}(\mathbf{k})$ is dominant by linear terms, $\boldsymbol{f}(\mathbf{k})$ can be written as

$$\boldsymbol{f}(\mathbf{k}) = \hat{M}\mathbf{k},\tag{4}$$

where $\hat{M}$ is a 3-by-3 matrix. According to Supplementary Eq. (3),

$$\hat{M}\mathbf{k} = \mathrm{Det}(R)R^{-1}\hat{M}R\mathbf{k}.\tag{5}$$

Hence

$$\hat{M} = \mathrm{Det}(R)R^{-1}\hat{M}R,\tag{6}$$

and

$$\mathrm{Det}(\hat{M}) = \mathrm{Det}(R)\mathrm{Det}(\hat{M}).\tag{7}$$

For achiral point groups $\mathcal{G}_{\mathbf{k}_0}$, there exists a roto-inversion operation $\tilde{R}$ with $\mathrm{Det}(\tilde{R}) = -1$, which further requires $\mathrm{Det}(\hat{M}) = 0$. Therefore, in an achiral point group, the determinant of $\hat{M}$ is always zero.

In the main text, we have assumed that the matrix $\hat{M}$ of an achiral point group is always diagonalizable, which can be verified by enumerating all possible forms of $\hat{M}$ for different achiral point groups (Supplementary Table 1). However, in some cases (C_{3v}, C_{4v} and C_{6v}), not all the eigen-values ϵ_j or eigen-vectors $\mathbf{n_j}$ of $\hat{M}$ are real. In spite of this fact, our argument in the main text still holds as the null eigen-vector $\mathbf{n_3}$ with eigen-value zero is always a real vector (multiplied by an overall trivial phase). This can be easily proved considering $\hat{M}$ is a real matrix.

D. KNLs enforced by roto-inversion symmetries: mirror, S_3 and S_4 symmetry

Let us further study the constraint of roto-inversion ($\mathrm{Det}(\tilde{R}) = -1$) on the specific form of $\boldsymbol{f}(\mathbf{k})$. For convenience, we use $k_{1,2}$ and k_3 to denote the coordinates perpendicular and parallel to the roto-inversion axis respectively. In general, a roto-inversion operation can be decomposed into a combination of an inversion I and a rotation C_n, *i.e.*

$$\tilde{R} = I\cdot C_n\tag{8}$$

Following Supplementary Eq. (3), the constraints of time-reversal symmetry and this roto-inversion symmetry impose

$$f_{\pm}(k_+,k_-,k_3) = -f_{\pm}(-k_+,-k_-,-k_3) = e^{\mp i\varphi}f_{\pm}(-e^{+i\varphi}k_+,-e^{-i\varphi}k_-,-k_3),\tag{9}$$

$$f_3(k_+,k_-,k_3) = -f_3(-k_+,-k_-,-k_3) = f_3(-e^{+i\varphi}k_+,-e^{-i\varphi}k_-,-k_3).\tag{10}$$

Here, $f_{\pm} = f_1 \pm i f_2$, $k_{\pm} = k_1 \pm i k_2$, and $\varphi = 2\pi/n$. With $f_{\pm}(\mathbf{k})$, the eigen-energies of $H(\mathbf{k})$ can be written as $E_{\pm}(\mathbf{k}) = f_0(\mathbf{k}) \pm \sqrt{f_+(\mathbf{k})f_-(\mathbf{k}) + f_3(\mathbf{k})^2}$. We should note here that the origin point of $\mathbf{k}$ vector in Supplementary Eq. (9) and Supplementary Eq. (10) is not necessary to be Γ but any TRIM with an achiral little group. In the following, we show that all roto-inversion symmetries mirror, S_3, S_4 enforce KNLs. (Note there does not contain S_6 in non-centrosymmetric achiral point group), where the S_n symmetry is defined as

$$S_n = m\cdot C_n,\tag{11}$$

representing the combination of a mirror and a n-fold rotation perpendicular to the mirror plane according to the Schoenflies notation. As a result, in Supplementary Eq. (8), $n = 6$ for S_3 and $n = 4$ for S_4.

(I) *For an achiral crystal with mirror symmetry, there always exist KNLs within the mirror plane.*

Before starting proceeding this part, we need to state two facts that if a crystal respects mirror symmetry m: (i) the set of primitive reciprocal lattice vectors can always be chosen in such a way that exactly two of them lies within any pre-chosen m-invariant k-plane. (ii) The m-invariant k-plane contains exactly four non-equivalent TRIMs, though some of them need not lie within the 1st Brillouin zone.

For a mirror symmetry ($n = 2$, $\varphi = \pi$, Supplementary

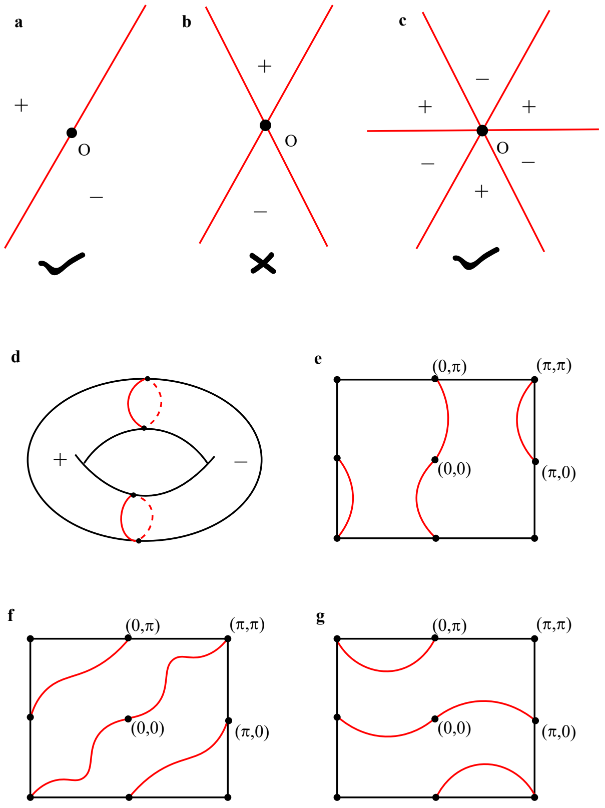

Supplementary Figure 1. KNLs within the mirror plane. **a**, **b**, **c** schematic plots of degenerate lines (red lines) coming from a TRIM within the mirror plane. $\pm$ here labels the sign of the scalar function $f_3(k_\parallel)$. **d** KNLs must completely cut the mirror-invariant Brillouin plane, which is topologically equivalent to a torus surface, into two separate parts. This requirement implies that there should be at least two KNLs within one mirror-invariant k plane. **e**, **f** and **g** Schematics for the three skeleton cases for KNLs within the mirror-invariant Brillouin plane, which are guaranteed by the time-reversal and a mirror symmetry.

Eq. (9) and Supplementary Eq. (10) yield

$$f_\pm(k_\parallel, k_3) = -f_\pm(-k_\parallel, -k_3) = -f_\pm(k_\parallel, -k_3), \quad (12)$$
$$f_3(k_\parallel, k_3) = -f_3(-k_\parallel, -k_3) = f_3(k_\parallel, -k_3). \quad (13)$$

where $k_\parallel = (k_1, k_2)$. Thus on the mirror-invariant k-planes where $k_3 = 0$ or π, $f_\pm$ terms vanish and the only finite f_3 term is odd in $k_\parallel$, *i.e.* $f_3(k_\parallel) = -f_3(-k_\parallel)$. Note any TRIM lying on the plane can be chosen as the origin point of $k_\parallel$.

Now the degenerate lines upon this plane are given by the equation

$$f_3(k_1, k_2) = 0. \quad (14)$$

And importantly, $f_3(k_1, k_2)$ is an odd (relative to any TRIM) scalar function defined on a 2D torus k-surface. Globally speaking, due to the odd function behavior of f_3, there exists at least one positive-valued area and correspondingly one negative-valued area, the boundaries of which give the KNLs and must pass through the TRIMs. KNLs emerging from TRIMs are thus actually protected by the odd property of f_3 as well as the topology of the k-surface. As the boundaries splitting the positive and negative areas, these KNLs have the following properties: (i) they have no end points; (ii) for a given TRIM, only an odd number of KNLs can come out (Supplementary Fig. 1**a**∼**c**); (iii) they must cut the torus k-surface into at least two separate parts, which implies that there are at least two KNLs for a mirror-invariant k-plane ((Supplementary Fig. 1**d**); (iv) they must connect two TRIMs. A time-reversal symmetric closed loop on the torus has to pass through two TRIMs (one is $k_\parallel = 0$, and the other is $k_\parallel \equiv -k_\parallel$ at the Brillouin zone boundary). Therefore, in the mirror plane, each degenerate line coming out from one TRIM has to connect with another TRIM, which forms the KNLs. Considering all these properties of the KNLs, the three simplest cases of how they should exist on the mirror-invariant k-plane is given in Supplementary Fig. 1**e**∼**g**, and all other more complicated cases are generated by adding more nodal lines to these three skeleton cases.

As shown above, the time-reversal and a mirror symmetry pin the KNLs upon the mirror-invariant k-plane, while allowing their tracks to go along quite arbitrary curves on the plane. However, additional crystal symmetries like C_2 and C_3 rotations can further constrain KNLs along some high-symmetry paths. We take a simple case as an illustration where the additional symmetry is a C_2 rotation whose rotation axis is within the mirror plane. Without loss of generality, the axis of C_2 can be set along the k_x-direction, *i.e.* C_{2x}. This new C_{2x} rotation requires the form of odd function $f_3(k_x, k_y)$ further satisfying $f_3(k_x, -k_y) = -f_3(k_x, k_y)$. Along the C_{2x} axis, $f_3(k_x, k_y = 0)$ vanishes and give rise to a straight KNL joining two TRIMs. Other additional symmetry cases can also be analyzed in this way, but a more systematic method to find all high-symmetry KNLs is to utilize the compatibility relation as we have presented in the main text.

(II) *For an achiral crystal with roto-inversion symmetry $S_3(n = 6)$ or $S_4(n = 4)$, there always exists a KNL along the $\langle 0, 0, k_3\rangle$-direction which is perpendicular to the roto-inversion plane.*

Along the $\langle 0, 0, k_3\rangle$-direction, Supplementary Eq. (9) and Supplementary Eq. (10) are simplified as

$$f_\pm(k_3) = -f_\pm(-k_3) = e^{\mp i\varphi} f_\pm(-k_3), \quad (15)$$
$$f_3(k_3) = -f_3(-k_3) = f_3(-k_3) \quad (16)$$

Hence, $f_\pm(\mathbf{k}), f_\pm(\mathbf{k})$ and $f_3(k_3)$ must vanish along the $\langle 0, 0, k_3\rangle$-direction when $(1 + e^{\mp i\varphi}) \neq 0$, which is the case for S_3 symmetry ($\varphi = \pi/3$) and S_4 symmetry ($\varphi = \pi/2$). In contrast for mirror symmetries with $\varphi = \pi$, we have $(1 + e^{\mp i\varphi}) = 0$, which allows finite $f_\pm$ that results in a finite splitting along the $\langle 0, 0, k_3\rangle$-direction.

Supplementary Note 3. KNLS FROM ACHIRAL LITTLE GROUPS BASED ON K· P ANALYSIS

To show the directions of KNLs coming from one TRIM explicitly, we derived the $\mathbf{k}\cdot\mathbf{p}$ Hamiltonians given by two dimensional double-valued irreducible representations (IRRs) for all non-centrosymmetric achiral little groups. In the Method Section of the main text, we have summarized these $\mathbf{k}\cdot\mathbf{p}$ Hamiltonians in Main text Table 2. Here, we summary Supplementary Table 1 to present more details including the principle axes, the specific bases. As shown in Supplementary Table 1, we further identified the touching types of KNLs given by each $\mathbf{k}\cdot\mathbf{p}$ Hamiltonian.

Generally, there are always KNLs emerging from TRIMs with achiral little groups. Notably, the features of KNLs of $J_z = \pm 1/2$ and $J_z = \pm 3/2$ fermions are different, where J_z is the z component of the total angular momentum. The way to identify whether a couple of bands belong to $J_z = \pm 1/2$ and $J_z = \pm 3/2$ fermions is by looking at how the states transform under rotational symmetry. By analyzing Supplementary Table 1, we find for $J_z = \pm 1/2$ fermions, there are KNLs within the mirror plane or along the roto-inversion axis of S3 and S4 symmetry, which is consistent with the general analysis given in Sec. II; while for $J_z = \pm 3/2$ fermions, KNLs are only enforced within the mirror plane. In Sec. V, we further show KNLs of $J_z = \pm 3/2$ fermions in a real material as an example.

In addition, it is also interesting to study the dispersion relation between the couple of bands around certain KNLs. By checking Supplementary Table 1, we find that besides linear touching KNLs, there are also quadratic KNLs in the C_{3h} and D_{3h} point groups and cubic KNLs in the C_{6v} point group.

Here the terms: linear, quadraticand cubic are defined by the dispersion of splitting between two bands upon a k-plane perpendicular to the KNLs that are studied. For a plane in the 3D Brillouin zone which intercepts a KNL, we can obtain a $\mathbf{k}\cdot\mathbf{p}$ Hamiltonian describing the states near the KNL on the momentum plane. For example, assuming a KNL along the k_z-direction, the $\mathbf{k}\cdot\mathbf{p}$ Hamiltonian near the KNL can be written as $H(\mathbf{p}) = f_0(\mathbf{p})\sigma_0 + v\mathbf{p}_+^m\sigma_+ + v\mathbf{p}_-^m\sigma_-$, where $\mathbf{p}$ denotes the momentum perpendicular to the KNL. Here, $\mathbf{p}_\pm = p_x + ip_y, \sigma_\pm = \sigma_x \pm \sigma_y$ and m=1,2,3 determines the energy dispersion of the states which gives linear-, quadratic- and cubic- energy dispersion respectively. We use the word *Dirac* to denote that case with $m = 1$ and the word *higher-order Dirac* to denote that cases with $m > 1$. Note that the Dirac point here is the interception point between the momentum plane and the KNL and it is always two-fold degenerate. By adiabatically moving an electron in a loop circling a given KNL, it will acquire a Berry phase of $m\pi$ mod 2π which can be experimentally probed by quantum oscillation.

Supplementary Note 4. DETERMINE KRAMERS NODAL LINES FROM COMPATIBILITY RELATIONS

In general when moving from a high symmetry point to a high symmetry line, the symmetry of k-points is reduced. For convenience, we denote the little group of the high symmetry point as $\mathcal{G}_1$, and its subgroup $\mathcal{G}_2$ as the little group of the high symmetry line. Then an irreducible representation Γ_1 of $\mathcal{G}_1$ can be decomposed as linear combinations of irreducible representations Γ_j of $\mathcal{G}_2$, i.e., the character χ of each unitary symmetry operation R satisfies

$$\chi(D_{\mathcal{G}_1}^{(\Gamma_1)}(R)) = \sum_j \chi(D_{\mathcal{G}_2}^{(\Gamma_j)}(R)), \tag{17}$$

This formula defines the compatibility relations. Here we illustrate how to determine Kramers nodal lines via compatibility relations with SG No. 156 ($P3m1$) as an example. The same method has been used to determine the nodal points and nodal lines enforced by non-symmorphic symmetries in Ref. [14–16].

For SG No. 156 ($P3m1$), relevant symmetries are the three-fold symmetry 3_{001} and the mirror symmetry m_{010} (k_xk_z-plane). The mirror plane contains TRIMs Γ, M, A, L and high symmetry lines Δ, U, R, Σ. Since the spin-orbital coupling is included, we need to consider double-valued representations, where a 2π rotation will yield a -1 phase. These double-valued irreducible representations are listed in Supplementary Table 2. At TRIMs, there is one additional requirement for irreducible representations: time-reversal invariance. The two-dimensional representations $\overline{\Gamma}_6$, $\overline{A}_6$ are pseudo-real, so they are time-reversal invariant by themselves [13]. All one-dimensional representations at TRIMs are complex and need to be paired up to form time-reversal-invariant representations or so-called co-representations [13]. With Supplementary Eq. (17), we are able to determine how these time-reversal invariant representations are split along high symmetry lines. The compatibility relations and the corresponding band connectivity are drawn in Supplementary Fig. 2. Evidently, only $\overline{\Gamma}_6$–$\overline{\Delta}_6$–$\overline{A}_6$ is able to support the two-fold degenerate KNL. Alternatively, one can identify this KNL by consulting the program DCOMPREL on Bilbao Crystallographic Server [17].

Here, we need to comment on a special case where degeneracy cannot be captured by the analysis of ordinary compatibility relations. When the high symmetry line considered is along a roto-inversion axis of S_n ($n = 4, 6$), such as in SG No. 174 ($P\bar{6}$) and SG No. 81 ($P\bar{4}$), a combined anti-unitary symmetry $\mathcal{T}S_n$ can also enforce degeneracy. Take SG No. 81 ($P\bar{4}$) as an example. The combined anti-unitary symmetry $\mathcal{T}S_4$ leaves the k-points upon Γ–Z (or equivalently high symmetry line Λ) invariant. Due to this anti-unitary symmetry, the double-valued complex irreducible representations $\overline{\Lambda}_3$ and $\overline{\Lambda}_4$ pair up and form a two-dimensional irre-

Supplementary Table 1. Kramers nodal lines (KNLs) from TRIMs with achiral little groups based on $\mathbf{k}\cdot\mathbf{p}$ Hamiltonian analysis. Here the point group denotes the one that ${}^{H}G^{\mathbf{k}}$ is isomorphic to, $k_\pm = k_x \pm ik_y$, and the Pauli matrices σ in the Table operate on the corresponding basis.

Point group	P-axes	Coreps	Basis	$\mathbf{k}\cdot\mathbf{p}$ Hamiltonian	Matrix $\hat{M}$	KNL	Touching
C_{1v}	$\hat{z}$ ($m \perp \hat{z}$)	$G_4^1 : R_2R_4$	$\|1/2, \pm 1/2\rangle$	$\alpha_{13}k_z\sigma_x + \alpha_{23}k_z\sigma_y +$<br>$(\alpha_{31}k_x + \alpha_{32}k_y)\sigma_z$	$\begin{pmatrix} 0 & 0 & \alpha_{13} \\ 0 & 0 & \alpha_{23} \\ \alpha_{31} & \alpha_{32} & 0 \end{pmatrix}$	$\in m$<br>$(-\alpha_{32}\hat{x} + \alpha_{31}\hat{y})$	linear
C_{2v}	$\hat{z}$	$G_8^5 : R_5$	$\|1/2, \pm 1/2\rangle$	$\alpha_{12}k_y\sigma_x + \alpha_{21}k_x\sigma_y$	$\begin{pmatrix} 0 & \alpha_{12} & 0 \\ \alpha_{21} & 0 & 0 \\ 0 & 0 & 0 \end{pmatrix}$	$\hat{z}$	linear
S_4	$\hat{z}$	$G_8^1 : R_2R_8$<br>$G_8^1 : R_4R_6$	$\|1/2, \pm 1/2\rangle$<br>$\|3/2, \pm 1/2\rangle$	$(\alpha_{11}k_x + \alpha_{12}k_y)\sigma_x +$<br>$(\alpha_{12}k_x - \alpha_{11}k_y)\sigma_y$	$\begin{pmatrix} \alpha_{11} & \alpha_{12} & 0 \\ \alpha_{12} & -\alpha_{11} & 0 \\ 0 & 0 & 0 \end{pmatrix}$	$\hat{z}$	linear
C_{4v}	$\hat{z}$	$G_{16}^{14} : R_7$;<br>$G_{16}^{14} : R_6$	$\|1/2, \pm 1/2\rangle$;<br>$\|3/2, \pm 1/2\rangle$	$\alpha_{12}k_y\sigma_x - \alpha_{12}k_x\sigma_y$	$\begin{pmatrix} 0 & \alpha_{12} & 0 \\ -\alpha_{12} & 0 & 0 \\ 0 & 0 & 0 \end{pmatrix}$	$\hat{z}$	linear
D_{2d}	$\hat{z}$	$G_{16}^{14} : R_7$;<br>$G_{16}^{14} : R_6$	$\|1/2, \pm 1/2\rangle$;<br>$\|3/2, \pm 1/2\rangle$	$\alpha_{11}k_x\sigma_x - \alpha_{11}k_y\sigma_y$	$\begin{pmatrix} \alpha_{11} & 0 & 0 \\ 0 & -\alpha_{11} & 0 \\ 0 & 0 & 0 \end{pmatrix}$	$\hat{z}$	linear
C_{3v}	$\hat{z}$	$G_{12}^4 : R_6$	$\|1/2, \pm 1/2\rangle$	$\alpha_{12}k_y\sigma_x - \alpha_{12}k_x\sigma_y$	$\begin{pmatrix} 0 & \alpha_{12} & 0 \\ -\alpha_{12} & 0 & 0 \\ 0 & 0 & 0 \end{pmatrix}$	$\hat{z}$	linear
C_{6v}	$\hat{z}$	$G_{24}^{11} : R_7, R_8$	$\|1/2, \pm 1/2\rangle$	$\alpha_{12}k_y\sigma_x - \alpha_{12}k_x\sigma_y$	$\begin{pmatrix} 0 & \alpha_{12} & 0 \\ -\alpha_{12} & 0 & 0 \\ 0 & 0 & 0 \end{pmatrix}$	$\hat{z}$	linear
T_d	$\hat{x}, \hat{y}, \hat{z}$	$G_{48}^{10} : R_4$;<br>$G_{48}^{10} : R_5$	$\|1/2, \pm 1/2\rangle$;<br>$\|3/2, \pm 1/2\rangle$	$\alpha(k_x(k_y^2 - k_z^2)\sigma_x + k_y(k_z^2 - k_x^2)$<br>$\sigma_y + k_z(k_x^2 - k_y^2)\sigma_z)$	—	$\hat{x}$, $\hat{y}$, $\hat{z}$<br>$\pm\hat{x} \pm \hat{y} \pm \hat{z}$	linear
C_{3v}	$\hat{z}$	$G_{12}^4 : R_3R_4$	$\|3/2, \pm 3/2\rangle$	$i\alpha_1(k_+^3 - k_-^3)\sigma_x + (\alpha_2k_z^3 +$<br>$\alpha_3k_+k_-k_z) + \alpha_4(k_+^3 + k_-^3))\sigma_y$<br>$+i\alpha_5(k_+^3 - k_-^3)\sigma_z$	—	$\in m$	linear
C_{3h}	$\hat{z}$	$G_{12}^1 : R_4R_{10}$;<br>$G_{12}^1 : R_6R_8$	$\|1/2, \pm 1/2\rangle$;<br>$\|3/2, \pm 1/2\rangle$	$(\beta_1k_+^2 + \beta_1^*k_-^2)k_z\sigma_x + i(\beta_1k_+^2$<br>$-\beta_1^*k_-^2)k_z\sigma_y + (\beta_2k_+^3 + \beta_2^*k_-^3)\sigma_z$	—	$\hat{z}$& $\in m$	quadratic
		$G_{12}^1 : R_2R_{12}$	$\|3/2, \pm 3/2\rangle$	$(\alpha_1k_z^3 + \alpha_2k_+k_-k_z)\sigma_x + (\alpha_3k_z^3$<br>$+\alpha_4k_+k_-k_z)\sigma_y + (\beta_1k_+^3 + \beta_1^*k_-^3)\sigma_z$	—	$\in m$	linear
D_{3h}	$\hat{z}$	$G_{24}^{11} : R_7$;<br>$G_{24}^{11} : R_8$	$\|1/2, \pm 1/2\rangle$;<br>$\|3/2, \pm 1/2\rangle$	$i\alpha_1(k_+^2 - k_-^2)k_z\sigma_x - \alpha_1(k_+^2$<br>$+k_-^2)k_z\sigma_y + i\alpha_2(k_+^3 - k_-^3)\sigma_z$	—	$\hat{x}$, $C_3\hat{x}$,$C_3^2\hat{x}$, $\hat{z}$	quadratic
		$G_{24}^{11} : R_9$	$\|3/2, \pm 3/2\rangle$	$(\alpha_1k_z^3 + \alpha_2k_+k_-k_z)\sigma_y +$<br>$+i\alpha_3(k_+^3 - k_-^3)\sigma_z$	—	$\hat{x}$, $C_3\hat{x}$,$C_3^2\hat{x}$	linear
C_{6v}	$\hat{z}$	$G_{24}^{11} : R_9$	$\|3/2, \pm 3/2\rangle$	$i\alpha_1(k_+^3 - k_-^3)\sigma_x +$<br>$+\alpha_2(k_+^3 + k_-^3)\sigma_y$	—	$\hat{z}$	cubic

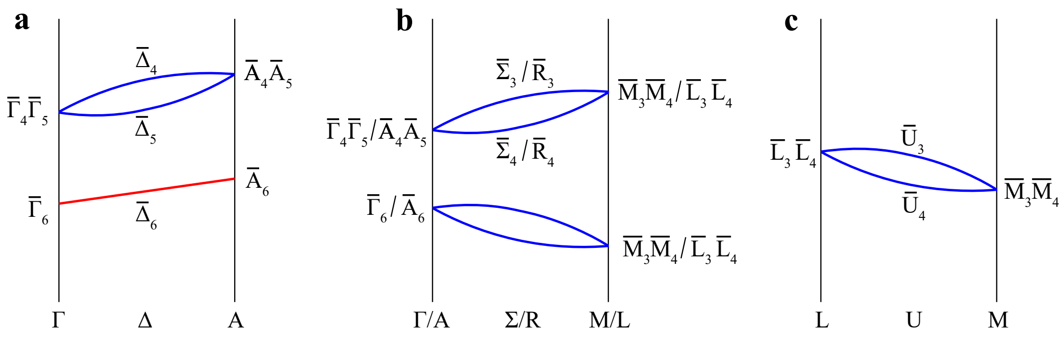

Supplementary Figure 2. Analysis of KNLs for SG No. 156 ($P3m1$) from compatibility relations. **a**, **b**, **c** demonstrate compatibility relations and band connectivity diagrams for SG No. 156, $P3m1$ along different k-path, inferred from Supplementary Table 2. High symmetry lines with two-fold degeneracy are highlighted in red color.

Supplementary Table 2. Double-valued irreducible representation (Irrep) of SG No. 156 ($P3m1$) at TRIMs Γ, A, M, L as well as high symmetry lines Δ, U, R, Σ. The notations follow from Ref. [17].

Irrep	3_{001}	m_{010}
$\bar{\Gamma}_4$	-1	$-i$
$\bar{\Gamma}_5$	-1	i
$\bar{\Gamma}_6$	$\begin{pmatrix} e^{-i\pi/3} & 0 \\ 0 & e^{i\pi/3} \end{pmatrix}$	$\begin{pmatrix} 0 & e^{-i\pi/3} \\ e^{-i2\pi/3} & 0 \end{pmatrix}$
$\bar{\Delta}_4$	-1	$-i$
$\bar{\Delta}_5$	-1	i
$\bar{\Delta}_6$	$\begin{pmatrix} e^{-i\pi/3} & 0 \\ 0 & e^{i\pi/3} \end{pmatrix}$	$\begin{pmatrix} 0 & e^{-i\pi/3} \\ e^{-i2\pi/3} & 0 \end{pmatrix}$
$\bar{A}_4$	-1	$-i$
$\bar{A}_5$	-1	i
$\bar{A}_6$	$\begin{pmatrix} e^{-i\pi/3} & 0 \\ 0 & e^{i\pi/3} \end{pmatrix}$	$\begin{pmatrix} 0 & e^{-i\pi/3} \\ e^{-i2\pi/3} & 0 \end{pmatrix}$
$\bar{L}_3$	–	$-i$
$\bar{L}_4$	–	i
$\bar{M}_3$	–	$-i$
$\bar{M}_4$	–	i
$\bar{U}_3$	–	$-i$
$\bar{U}_4$	–	i
$\bar{\Sigma}_3$	–	$-i$
$\bar{\Sigma}_4$	–	i
$\bar{R}_3$	–	$-i$
$\bar{R}_4$	–	i

ducible co-representation, which yields the Γ–Z KNL in SG No. 81 ($P\bar{4}$). In this case, we can also understand this KNL from the eigenvalue method [14]. The Hamiltonian H along Γ–Z in SG No. 81 ($P\bar{4}$) is actually not only invariant under the $\mathcal{T}S_4$ operation, but also under the C_2 operation. Let us consider a simultaneous eigenstate of H and C_2 as ψ, with $C_2\psi = \lambda\psi$ and $H\psi = E\psi$. It is easy to show $\mathcal{T}S_4\psi$ is also an eigenstate of C_2 with eigenvalue λ^* as well as an eigenstate of H with eigenvalue E, because we have $[\mathcal{T}S_4$ and $C_2] = 0$ and $[\mathcal{T}S_4, H] = 0$. The 1/2 spin of electrons further requires $\lambda^2 = -1$, leading to $\lambda = \pm i$ and $\lambda = -\lambda^*$. This means ψ and $\mathcal{T}S_4\psi$ are two distinct states with the same eigen-energy E and they form a two-dimensional irreducible co-representation.

The derivation of this special case for other space groups listed in main text Table 1 proceeds in a similar way. All of the allowed KNLs by the space group symmetries are summarized in main text Table 1. They are compatible with the $\mathbf{k}\cdot\mathbf{p}$ analysis given in Supplementary Table 1.

Supplementary Note 5. TRIMS IN ACHIRAL CRYSTALS

A. An overview of symmorphic space groups

There are in total 73 symmorphic space groups. Among these 73 space groups, there are 21 centrosymmetric space space groups: C_i: 2; C_{2h}: 10 and 12; D_{2h}: 47, 65, 69 and 71; C_{4h}: 83 and 87; D_{4h}: 123 and 139; C_{3i}: 147 and 148; C_{6h}: 175; D_{6h}: 191; T_h: 200 and 202, 204; O_h:221, 225 and 229;

27 non-centrosymmetric chiral space groups: C_1: 1; C_2: 3 and 5; D_2: 16, 21, 22 and 23; C_4: 75 and 79; D_4: 89 and 97; C_3: 143 and 146; D_3: 149, 150 and 155; D_{3d}: 162, 164 and 166; C_6: 168; D_6: 177; T : 195, 196 and 197; O: 207, 209 and 211;

and 25 non-centrosymmetric achiral space groups: C_{1h}: 6 and 8; C_{2v}: 25, 35, 38, 42 and 44; S_4: 81 and 82; C_{4v}: 99 and 107; D_{2d}: 111, 115, 119 and 121; C_{3v}: 156, 157 and 160; C_{3h}: 174; C_{6v}: 183; D_{3h}: 187 and 189; T_d: 215, 216 and 217.

B. Little groups of TRIMs in achiral space groups

In the main text, Type I and Type II KNLMs are identified according to the little groups of TRIMs. Here,

Supplementary Table 3. The little groups of TRIMs in non-centrosymmetric achiral symmorphic space groups.

SG No.	TRIMs with achiral little group	TRIMs with chiral little group
6,Pm	Γ, B, Y, A, Z, C, D and E are all C_{1h}.	–
8,Cm	Γ, Y, A and M are all C_{1h}.	–
25,$Pmm2$	Γ, Z, Y, T, X, U, S and R are all C_{2v}.	–
35,$Cmm2$	Γ, Z, Y and T are all C_{2v}.	S and R are both C_2.
38,$Amm2$	Γ, Y, T, Z and are all C_{2v}.	–
42,$Fmm2$	Γ, Z, T and Y are all C_{2v}.	–
44,$Imm2$	Γ and X are C_{2v}; S and R are C_{1v}.	T is C_2.
81,$P\bar{4}$	Γ, Z, M and A are all S_4	X and R are both C_2.
82,$I\bar{4}$	Γ, M are both S_4.	N is C_1, X is C_2.
99,$P4mm$	Γ, Z, M and A all are C_{4v};X and R are both C_{2v}.	–
107,$I4mm$	Γ and M are both C_{4v}; X is C_{2v}; N is C_{1v}.	–
111,$P\bar{4}2m$	Γ, A, Z and M are all D_{2d}.	X and R are both D_2.
115,$P\bar{4}m2$	Γ, M, A and Z are all D_{2d}; X and R are both C_{2v}.	–
119,$I\bar{4}m2$	Γ and M are both D_{2d}, N is C_{1v}.	X is D_2.
121,$I\bar{4}2m$	Γ and M are both D_{2d}, X is C_{2v}.	N is C_2.
156,$P3m1$	Γ and A are both C_{3v}; M and L are C_{1v}.	–
157,$P31m$	Γ and A are both C_{3v}; M and L are both C_{1v}.	–
160,$R3m$	Γ and T are both C_{3v}; L and FA are both C_{1v}.	–
174,$P\bar{6}$	Γ and A are both C_{3h}; M and L are both C_{1v}.	–
183,$P6mm$	Γ and A are both C_{6v}; M and L are both C_{2v}.	–
187,$P\bar{6}m2$	Γ and A are both D_{3h}; M and L are both C_{2v}.	–
189,$P\bar{6}2m$	Γ and A are both D_{3h}; M and L are both C_{2v}.	–
215,$P\bar{4}3m$	Γ and R are both T_d; M and X are both D_{2d}.	–
216,$F\bar{4}3m$	Γ is T_d; X is D_{2d}; L is C_{3v}.	–
217,$I\bar{4}3m$	Γ and H are both T_d.	–

we summarize the little groups of all TRIMs in non-centrosymmetric achiral symmorphic space groups in Supplementary Table 3. The little group of each TRIM can be identified by consulting the program KVEC and MKVEC on the Bilbao Crystallographic Server [17–19]. Apparently, among the 25 non-centrosymmetric symmorphic achiral space groups, seven space groups (SG No. 35, 44, 81, 82, 111, 119 and 121) support chiral TRIMs, while the other eighteen ones do not. As discussed in the main text, these chiral TRIMs that host electronic states described by a two-dimensional irreducible corepresentations of their little groups will emerge as Kramers Weyl points in achiral crystals. Notably, the appearance of Kramers Weyl points at high-symmetry points in achiral crystals was introduced in Ref. [20] as well.

Supplementary Note 6. MODEL HAMILTONIANS FOR RASHBA SEMICONDUCTOR BITEI AND THE CIRCULAR PHOTOGALVANIC EFFECT IN STRAINED BITEI

A. A four-band effective low energy model for BiTeI

Here, we derive the model Hamiltonians of BiTeI that are used in the main text. The lattice structure of BiTeI belongs to SG No. 156 ($P3m1$), the point group of which is a polar point group C_{3v} which is generated by a three-fold rotation C_3 along the z axis (*i.e.* 3_{001}) and vertical mirror symmetry σ_v (*i.e.* m_{010}). According to the *ab initio* method, the bands lying closest to Fermi energy are $|\Lambda, p_z, J_z = \pm 1/2\rangle$ bands, where Λ=Bi,Te,I [21]. By analyzing the transformation properties of $|\Lambda, p_z, \pm 1/2\rangle$, the four bands near Fermi energy at A point are found to belong to the spinor irreducible representation $\overline{\Gamma}_6$ of double group C_{3v}.

Supplementary Table 4. Model parameters for effective Hamiltonian 18. The chemical potential μ is chosen to be near the crossing point of the conduction band.

C_0^c(eV)	C_1^c(eV·Å^2)	C_2^c(eV·Å^2)	α_0^c(eV·Å)	α_1^c(eV·Å^3)
0.2491	24.0587	3.2389	1.4687	-18.2993
C_0^v(eV)	C_1^v(eV·Å^2)	C_2^v(eV·Å^2)	α_0^v(eV·Å)	α_1^v(eV·Å^3)
0.0757	-4.4977	-7.7134	-0.0982	-0.5719
$\mathcal{M}_0^0$ (eV)	$\mathcal{M}_1^0$(eV·Å)	$\mathcal{M}_2^0$(eV·Å^2)	$\mathcal{A}^0$(eV·Å)	$\mathcal{B}^0$(eV·Å)
0.2362	-6.6320	2.5584	0.3689	2.3023
$\mathcal{D}^0$(eV·Å^2)	μ(eV)	a (Å)	c (Å)	
1.4725	0.1151	4.425	7.378	

Based on this symmetry analysis, we can construct a four-band low energy effective Hamiltonian:

$$H_{eff}(\mathbf{k}) = \begin{pmatrix} \epsilon_1(\mathbf{k}) & V_0(\mathbf{k}) \\ V_0^\dagger(\mathbf{k}) & \epsilon_2(\mathbf{k}) \end{pmatrix}. \tag{18}$$

Here

$$\epsilon_i(\mathbf{k}) = C_0^i + C_1^i k_\parallel^2 + C_2^i k_z^2 \\ + (\alpha_0^i + \alpha_1^i k_\parallel^2)(k_x\sigma_y - k_y\sigma_x) + \beta^i k_y(3k_x^2 - k_y^2)\sigma_z \tag{19}$$

$$V_0(\mathbf{k}) = \mathcal{M}_0 + \mathcal{M}_1 k_\parallel^2 + \mathcal{M}_2 k_z^2 - i\mathcal{A}k_z \\ + \mathcal{B}(k_x\sigma_y - k_y\sigma_x) - i\mathcal{D}((k_x^2 - k_y^2)\sigma_y + 2k_xk_y\sigma_x) \tag{20}$$

with $k_\parallel^2 = k_x^2 + k_y^2$. The values of these parameters can be determined by fitting the unstrained BiTeI DFT band structure, which are listed in Supplementary Table 4.

B. The circular photogalvanic effect of strained BiTeI

The circular photogalvanic effect (CPGE) describes the DC part of the photocurrent produced by circularly polarized light which reverses sign when circular polarization is reversed. The quantization of CPGE is a signal for the emergence of Kramers Weyl points [20]. The the chiral charge $\mathcal{C}$ shown in main text Fig.4 is defined as [22, 23]

$$\mathcal{C} = \mathrm{Tr}(\beta)/i\beta_0 \tag{21}$$

with CPGE tensor

$$\beta_{ij}(\omega) = \frac{\pi e^3}{\hbar V}\epsilon_{ijk}\sum_{\mathbf{k},n,m} f_{nm}^{\mathbf{k}}\Delta_{\mathbf{k},nm}^i r_{\mathbf{k},nm}^k r_{\mathbf{k},mn}^l \delta(\hbar\omega - E_{\mathbf{k},mn}). \tag{22}$$

Here, $\beta_0 = \pi e^3/h^2$, V is the sample volume, $r_{\mathbf{k},nm} = i\langle n|\partial_{\mathbf{k}}|m\rangle$ is the Berry connection between the nth and mth bands, $E_{\mathbf{k},nm} = E_{\mathbf{k},n} - E_{\mathbf{k},m}$, $f_{nm}^{\mathbf{k}} = f_n^{\mathbf{k}} - f_m^{\mathbf{k}}$ represent the energy difference and Fermi-Dirac distribution, respectively, and $\Delta_{\mathbf{k},nm}^i = \partial_{\mathbf{k}_i}E_{\mathbf{k},mn}/\hbar$ is the electron velocity. From this formula, we calculated the trace of CPGE tensor of a strained BiTeI, which is captured by the Hamiltonian $H_{eff}(\mathbf{k}) + H_{strain}$. The strained effects are described by a mirror-broken phenomenological Hamiltonian $H_{strain} = \lambda k_z\sigma_z$. We estimate the value of λ by fitting the splitting of Γ–Z in a strained band structure with $\lambda = 5$ meV, 15meV and 25 meV for 1%, 3% and 5% strains, respectively. The Fig. 4 in the main text was calculated using this model, and the chemical potential has been set near the Weyl nodes of the conduction band. Note that the influence of valence bands on the CPGE has also been taken into consideration in this four-band model, while we found that this influence is actually negligible within the low frequency region, which is consistent with the result in Ref. [22].

Supplementary Note 7. MORE REPRESENTATIVE MATERIALS OF KNLMS

In this section, we list some representative materials of KNLMs, including some special cases which have not been discussed in the main text.

A. $BiPd_2Pb$: C_{1v} system with only one mirror

The band structure of $BiPd_2Pb$ (SG No. 8, Cm, point group C_{1v}) is shown in Supplementary Fig. 3**c**. The two bands depicted in red are used to plot Fig. 2**j** of the main text. Unlike the cases of most of the material listed in main text Table 1, where all KNLs are pinned along high-symmetry paths by the crystal symmetry, the single mirror symmetry in C_{1v} only restricts the KNLs to lie within the mirror-invariant k-plane but not necessarily along high-symmetry paths, as shown in Supplementary Fig. 3**b** and Supplementary Fig. 3**c**. A more clear figure of the KNLs which is directly related to the DFT results has been presented in the main text Fig. 2.

B. $CsPbF_3$: A Nonsymmorphic KNLM

In the main text, we have constrained our discussion within the symmorphic crystals as some non-symmorphic symmetry could give rise to nodal planes on the Brillouin zone boundaries. In the following, a non-symmorphic KNLM $CsPbF_3$ is given as an example to illustrate that our discussion can also be applied to non-symmorphic crystals. $CsPbF_3$ belongs to the non-symmorphic SG No. 161 which is also denoted as $\Gamma_{rh}C_{3v}^6$ or $R3c$. As shown in Supplementary Fig. 3**e**, and Supplementary Fig. 3**f**, there are KNLs along the Γ–Z path in $CsPbF_3$, which is similar to the case of symmorphic SG No. 160 ($R3m$) shown in the main text Table 1. In addition, there is also a KNL connecting TRIMs Z and L via point B/B_1, which is denoted by the green lines in the corresponding figures. It should be noted that due to the lack of screw symmetries in SG No. 161 ($R3c$), there is no non-symmorphic symmetry forcing nodal planes on the Brillouin zone boundary for the case of $CsPbF_3$, which was verified by DFT calculations. As discussed in Ref. [20], nodal degeneracies at the zone-boundary $k_i = \pi/a_i$ can be supported if the screw symmetry $\{C_{2,i}|\mathbf{t}\}$ with $\mathbf{t}_i = a_i/2$ (a_i as lattice constant along i axis) being contained in the SG symmetry G. This is because a combined anti-unitary symmetry $\{C_{2,i}|\mathbf{t}\}\mathcal{T}$ can be defined at the Brillouin zone boundary $k_i = \pi/a_i$, which leaves $\mathbf{k}$ to be invariant at this plane and

$$(\{C_{2,i}|\mathbf{t}\}\mathcal{T})^2 = e^{-i(\frac{\hat{k}_i a_i}{2} + \frac{\hat{k}_j a_j}{2})}C_{2,i}\mathcal{T}e^{-i(\frac{\hat{k}_i a_i}{2} + \frac{\hat{k}_j a_j}{2})}C_{2,i}\mathcal{T} \\ = e^{-ik_i a_i} = -1. \tag{23}$$

Here, the index j labels the other two components that are orthogonal to k_i, the crystal momentum operator re-

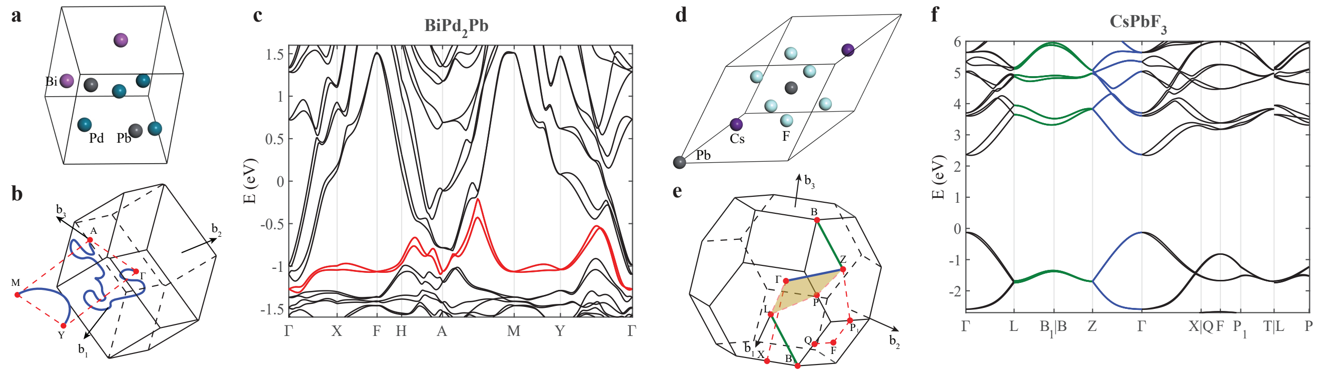

Supplementary Figure 3. Example materials of crystals with the C_{1v} and nonsymmorphic symmetry. **a** and **b** show the primitive cell and the first Brillouin zone of $BiPd_2Pb$, respectively. The DFT calculated $BiPd_2Pb$ band structures is presented in **c**. The degenerate KNLs given by the two red-colored bands in **c** are plotted as blue curves in **b**. **d, e** and **f** respectively show the non-symmorphic material $CsPbF_3$'s primitive lattice cell, first Brillouin zone and DFT bands, respectively. The thick blue and green curves in **e** and **f** are the KNLs connecting Γ-T and T-L, where KNL L-B-T enforced by the glide mirror symmetry is highlighted as green color.

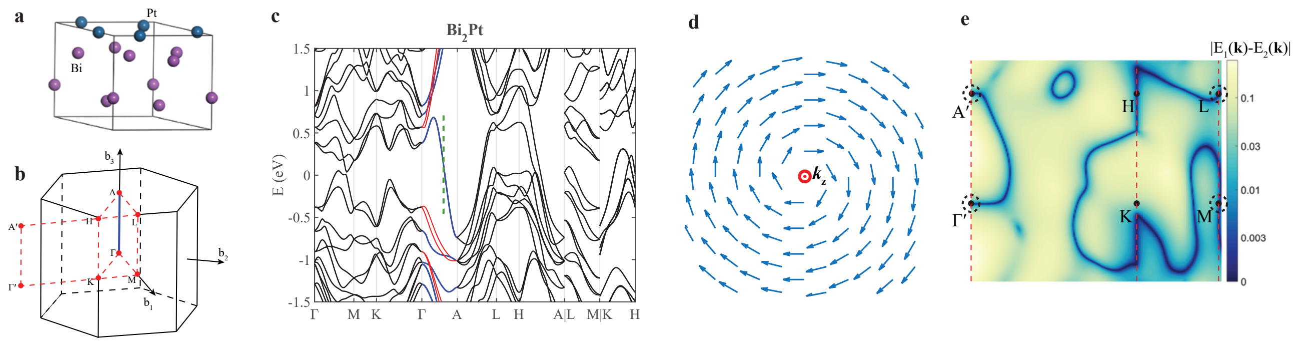

Supplementary Figure 4. Bi_2Pt (SG No. 157, $P31m$): a KNLM with $J_z = \pm 3/2$ bands. **a, b** and **c** show Bi_2Pt's primitive lattice cell, 1st Brillouin zone and its DFT bands, respectively. The thick blue and red curves along the Γ-A line in **c** represent $J_z = \pm 1/2$ and $J_z = \pm 3/2$ bands, respectively. **d** plots the DFT-obtained spin texture on a plane (denoted by the green dashed line in **c**) perpendicular to the KNL Γ–A. **e** plots the band gap of two Bi_2Pt's $J_z = \pm 3/2$ bands on the mirror-invariant plane upon which contains four TRIMs, Γ′, A′, L and M. Note that H and K are two high symmetry points but not TRIMs on the mirror plane. The mirror plane is illustrated in **b**. Two KNLs connecting Γ′–A′ and M–L can be easily identified.

spects $\mathcal{T}\hat{k}_i\mathcal{T}^{-1} = -\hat{k}_i$. These additional nodal degeneracies at $k_i = \pi/a_i$ overwhelm the KNLs on this plane.

Although the glide symmetry is not related to the nodal plane degeneracies at Brillouin zone boundaries, we found it can enforce some extra KNLs at Brillouin zone boundaries. Especially, the direction of the KNL is perpendicular to the glide mirror plane in this case. To be specific, we consider a glide mirror symmetry $\{m_1|\mathbf{t}\}$, where the translational operation $\mathbf{t} = (a_1/2, a_2/2, a_3/2)$ with $a_{2,3}/2(a_1/2)$ as the translations within along (perpendicular to) the mirror plane, and the Brillouin zone boundary is taken as $k_3 = \pi/a_3$. Then we show the combined symmetry $\{m_1|\mathbf{t}\}\mathcal{T}$ is a well-defined anti-unitary symmetry for the line $\mathbf{k} = (k_1, 0, \pi/a_3)$, which lies at the Brillouin zone boundary $k_3 = \pi/a_3$ and is perpendicular to the glide mirror plane. It can be noted that this line is invariant under this combined symmetry operation $\{m_1|\mathbf{t}\}\mathcal{T}$ and on this line, the square of $\{m_1|\mathbf{t}\}\mathcal{T}$ is given by

$$(\{m_1|\mathbf{t}\}\mathcal{T})^2 = e^{-i(k_2a_2+k_3a_3)}m_1^2\mathcal{T}^2 = -1 \qquad (24)$$

Hence, we can see that a glide mirror symmetry enforces a degenerate line that is perpendicular to the glide mirror plane at the Brillouin zone zone boundary for non-magnetic crystals. Indeed, the KNL connecting TRIMs Z and L via point B/B_1 shown in Supplementary Fig. 3**e**, and Supplementary Fig. 3**f** is enforced by the glide mirror symmetry that is along Γ–Z and perpendicular to Z–B direction. Notice that the nodal line Z–B can be extended to the KNL Z–L and part of the KNL Z–L are folded back on the Brillouin zone boundary as B_1–L.

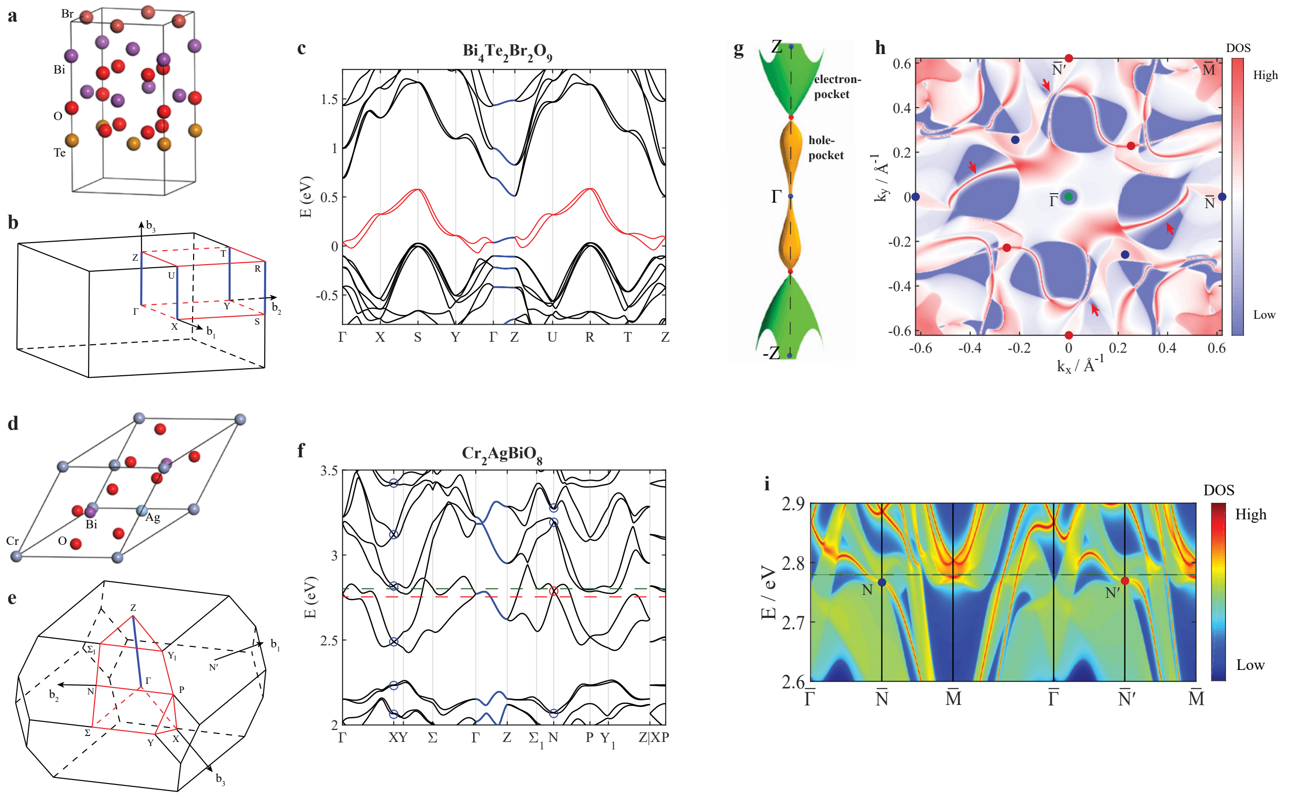

Supplementary Figure 5. Example materials of type I and type II KNLM with the octdong Fermi surface. In **a, b** and **c** we show the primitive lattice cell, the first Brillouin zone and the conduction bands, respectively, of the KNLM, $Bi_4Te_2Br_2O_9$, which possesses octdong Fermi surfaces as mentioned in the main text Sec. II C. The KNLs along Γ–Z are denoted by thick blue lines in both **b** and **c**. In **d, e** and **f** we illustrate the primitive lattice cell, the first Brillouin zone and the conduction bands of the Type II KNLM Cr_2AgBiO_8. As a Type II KNLM, Cr_2AgBiO_8 has Kramers Weyl points N (N′) and X, which are circled in **f**. In **g**, the octdong-type Fermi surface resulting from the Γ–Z KNL are drawn at $E_F = 2.768$eV, as denoted by the red dashed line in **f** (some trivial Fermi surfaces near this octdong Fermi surface are not depicted in **g**). Here the red dots denote the touching points of the electron pocket centered at Z and the hole pocket centered at Γ. In **h**, we plot the (001) surface spectral function of Cr_2AgBiO_8 at $E = 2.78$eV (denoted as a green dashed line in both **f** and1 **i**). The blue (red) solid dots represent the Weyl points with negative (positive) chiral charges. Note that the Kramers Weyl point N (N′) projects to $\bar{\text{N}}$ ($\bar{\text{N}}'$) on the surface Brillouin zone. The four Fermi arcs originating from the N and N′ pockets are pointed out by red arrows. **i** is the surface spectral function along a k-path on the surface Brillouin zone.

C. Bi_2Pt: KNLM with $J_z = \pm 3/2$ bands

Under most circumstances, we have assumed that the electronic states transform as $|J = \frac{1}{2}, J_z = \pm\frac{1}{2}\rangle$ states under symmetry operations. However, as is shown in Supplementary Table 1, it is possible for some point groups (C_{3v}, C_{3h}, D_{3h} and C_{6v}) to have double-valued IRs corresponding to $|J = \frac{3}{2}, J_z = \pm\frac{3}{2}\rangle$ states. Although $|J = \frac{3}{2}, J_z = \pm\frac{3}{2}\rangle$ states transform under mirror m_z the same way as $|J = \frac{1}{2}, J_z = \pm\frac{1}{2}\rangle$ states, leading to the same conclusion that there are KNLs in the mirror-invariant plane, they behave quite differently under roto-inversion $S_3 = m_z C_3 = IC_6$ with $\varphi = \pi/3 \cdot 3 = \pi$, as defined in Supplementary Eq. (9) and Supplementary Eq. (10). By applying Supplementary Eq. (15), we find that a finite $f_\pm$ is allowed upon the roto-inversion axis, in contrast to the case of $|J = \frac{1}{2}, J_z = \pm\frac{1}{2}\rangle$, where along this axis lies a KNL.

In Supplementary Fig. 4**c** we give a vivid example for the above discussion by showing the DFT band structure of Bi_2Pt (SG No. 157, $P31m$, point group C_{3v}). In the spectrum, the red curves that split along the Γ-A line represent the $J_z = \pm 3/2$ bands, while blue curves, which are doubly-degenerate KNLs along Γ-A, belong to the $J_z = \pm 1/2$ bands. Although the KNLs of $J_z = \pm 3/2$ bands are not along the high-symmetry paths, they still exist in the mirror-invariant planes as shown in Supplementary Fig. 4**e**, which is consistent with the prediction made in Supplementary Note 3.

Supplementary Table 5. The symmetry-allowed higher-dimensional corepresentations at TRIMs for non-magnetic non-centrosymmetric achiral crystals.

Symmorphic SGs							
SG No.	TRIMs	AGs and coreps under $\mathcal{T}$	d	SG No.	TRIMs	AGs and coreps under $\mathcal{T}$	d
215 $F\bar{4}3m$ (T_d)	Γ, R	G_{48}^{10}: R_8	4	216 $F\bar{4}3m$ (T_d)	Γ	G_{48}^{10}: R_8	4
217 $I\bar{4}3m$ (T_d)	Γ, H	G_{48}^{10}: R_8	4				
**Nonsymmorphic SGs**							
SG No.	TRIMs	AGs and coreps under $\mathcal{T}$	d	SG No.	TRIMs	AGs and coreps under $\mathcal{T}$	d
26 $Pmc2_1$, 27 $Pcc2$ (C_{2v})	Z,U,T	G_{16}^{8}: R_9R_9	4	29 $Pca2_1$ (C_{2v})	Z, U	$G_{16}^{8} : R_9R_9$	4
30 $Pnc2$, 31 $Pmn2_1$ (C_{2v})	Z, U	$G_{16}^{8} : R_9R_9$	4	32 $Pba2$ (C_{2v})	S, R	$G_{16}^{8} : R_9R_9$	4
33 $Pna2_1$ (C_{2v})	Z, S, R	$G_{16}^{8} : R_9R_9$	4	34 $Pnn2$ (C_{2v})	Z, S	$G_{16}^{8} : R_9R_9$	4
36 $Cmc2_1$ (C_{2v})	Z, T	$G_{16}^{8} : R_9R_9$	4	37 $Ccc2$ (C_{2v})	Z, T	$G_{16}^{8} : R_9R_9$	4
43 $Fmm2$ (C_{2v})	Z	$G_{16}^{8} : R_9R_9$	4	101 $P4_2cm$ (C_{4v})	R	$G_{16}^{8} : R_9R_9$	4
103 $P4cc$ (C_{4v})	Z, A	$G_{32}^{10} : R_6R_6, R_7R_7$	4	103 $P4cc$ (C_{4v})	R	$G_{16}^{8} : R_9R_9$	4
104 $P4nc$ (C_{4v})	Z	$G_{32}^{10} : R_6R_6, R_7R_7$	4	106 $P4_2bc$ (C_{4v})	A	$G_{32}^{10} : R_6R_6, R_7R_7$	4
114 $P\bar{4}2_1c$ (D_{2d})	A	$G_{32}^{10} : R_6R_6, R_7R_7$	4	116 $P\bar{4}c2$ (D_{2d})	R	$G_{16}^{8} : R_9R_9$	4
159 $P31c$ (C_{3v})	A	$G_{12}^{4} : R_5R_5$	4	161 $R3c$ (C_{3v})	Z	$G_{12}^{4} : R_5R_5$	4
184 $P6cc$ (C_{6v})	L	$G_{16}^{8} : R_9R_9$	4	184 $P6cc$ (C_{6v})	A	$G_{48}^{12} : R_7R_7, R_8R_8, R_9R_9$	4
185 $P6_3cm$, 186 $P6_3mc$ (C_{6v})	L; A	$G_{16}^{8} : R_9R_9$;$G_{48}^{13} : R_{14}R_{15}$	4	188 $P\bar{6}c2$ (D_{3h})	A	$G_{48}^{14} : R_{11}R_{12}$	4
190 $P\bar{6}2c$ (D_{3h})	A	$G_{48}^{14} : R_{11}R_{12}$	4	218 $P\bar{4}3n$ (T_d)	Γ; X	$G_{48}^{10} : R_8$; $G_{32}^{11} : R_6R_7$	4
218 $P\bar{4}3n$ (T_d)	R	$G_{96}^{7} : R_6R_7$	4	219 $F\bar{4}3c$ (T_d)	Γ	$G_{48}^{10} : R_8$	4
220 $I\bar{4}3d$ (T_d)	Γ	$G_{48}^{10} : R_8$	4	220 $I\bar{4}3d$ (T_d)	H	$G_{96}^{7} : R_6R_7$	4
218 $P\bar{4}3n$ (T_d)	R	$G_{96}^{7} : R_{15}R_{15}$	8	220 $I\bar{4}3d$ (T_d)	H	$G_{96}^{7} : R_{15}R_{15}$	8

D. $Bi_4Te_2Br_2O_9$: Type I KNLM with the octdong Fermi surface

In the main text, we have already mentioned the Type I KNLM $Bi_4Te_2Br_2O_9$ (SG No. 25, $Pmm2$) with four separate KNLs. In Supplementary Fig. 5**c**, we further show its DFT band structures, within which the couple of bands represented by red curves are the ones related to the octdong Fermi surfaces plotted in the main text Fig. 3.

E. Cr_2AgBiO_8: Type II KNLM with the octdong Fermi surface

Cr_2AgBiO_8 (SG No. 82, $I\bar{4}$) is a Type II KNLM as listed in main text Table 1, bearing Kramers Weyl points located at X and N (N′). Among all the listed Type II KNLMs, this material specially attracts our interest, because all its bands near the Fermi energy are quite flat compared to its huge SOC splitting, as shown in Supplementary Fig. 5**f**. This feature provides us with an opportunity to observe the octdong Fermi surface and the Fermi arcs originating from the Kramers Weyl points in this Type II KNLM.

Its KNL is along Γ–Z, as shown in Supplementary Fig. 5**e**. By setting the Fermi energy across a KNL (*i.e.* the red dashed line in Supplementary Fig. 5**f**), the rare octdong Fermi surface resulting from the Γ–Z KNL can be seen (Supplementary Fig. 5**g**).

To show the Fermi arc states, we calculated the surface spectral function at the energy level near $E(\mathbf{k} = \mathrm{N})$ with the surface normal vector parallel to Γ–Z (Supplementary Fig. 5**h** and Supplementary Fig. 5**i**). Along this projection direction, two distinct N (N′) points carrying chiral charge $C = -1$ (+1) each, are projected onto the same surface N̄ (N̄′) point. This gives rise to two time-reversal related Fermi arcs coming out from the surface N̄ (N̄′) point (as pointed out by the red arrows in Supplementary Fig. 5**h**). Similar to the chiral KWSs, the Fermi arcs in Type II KNLMs are exceptionally long, spanning the entire Brillouin zone as the Kramers Weyl points are well separated in the reciprocal space. Through this example, we demonstrate that the Fermi arcs originating from the Kramers Weyl points are allowed not only in chiral KWSs, but also in achiral Type II KNLMs.

F. The higher-dimensional corepresentations at TRIMs for non-magnetic non-centrosymmetric achiral crystals

As discussed in main text, there allows higher dimensional corepresentations in some cases. To identify the feature of KNLs in these cases, in this section, we summary symmetry-allowed higher-dimensional corepresentations at TRIMs for non-magnetic non-centrosymmetric achiral crystals (Supplementary Table 5) and present some realistic material examples that support higher dimensional corepresentations.

We explicitly enumerated the possible higher-dimensional corepresentations, which are labeled with the irreducible of abstract groups, allowed by the Herring's little group ${}^{H}G^{\mathbf{k}}$ at TRIMs (c.f. [13]). The results are summarized in Supplementary Table 5. In symmor-

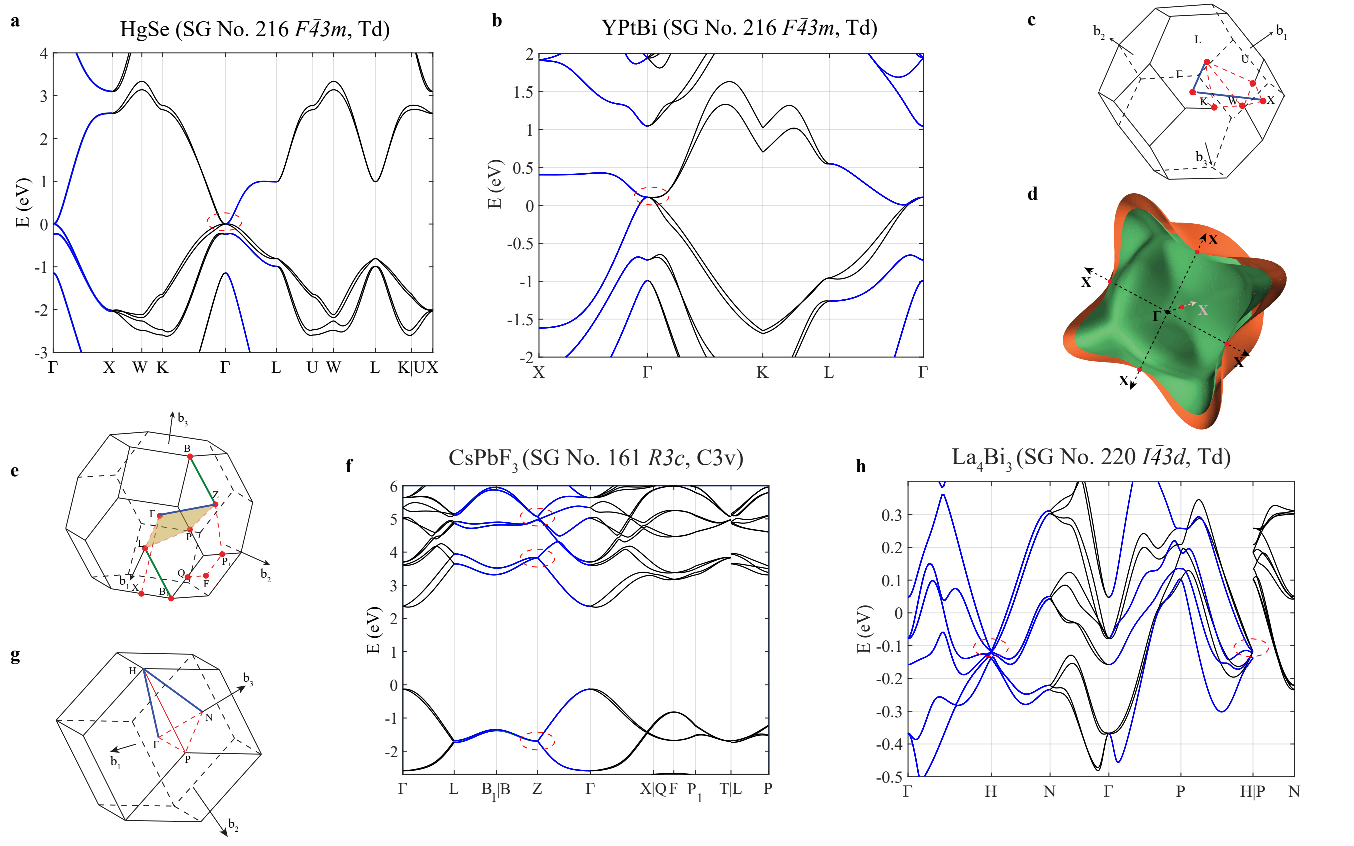

Supplementary Figure 6. Example materials that exhibit higher copres near Fermi level. **a** and **b**, respectively, show the band structures of symmorphic crystals HgSe (SG No. 216 F$\bar{4}$ 3m,T_d), half-Heuser material YPtBi (SG No. 216 F$\bar{4}$3m,T_d). **c** and **d**, respectively, show the the 1st Brillouin zone of HgSe, YPtBi and Fermi surface of HgSe at $E = -0.3$eV upon which the red dots denote the touching points. **e**, **g** and **f**, **h**, respectively, show the 1st Brillouin zone and the band structures of nonsymmorphic crystals $CsPbF_3$ (SG No. 161 R3c, C_{3v}), La_4Bi_3 (SG No. 220 I$\bar{4}$3d, T_d). The energy bands at TRIMs described by 4D corepresentations are circled in (**a**,**b**,**f**) and described by 8D corepresentations are circled in **h**. And KNLs are depicted with blue color in band structures and Brillouin zones

phic groups, there allows 4D corepresentations in TRIMs respecting T_d symmetry, including TRIMs Γ, R in SG No. 215 ($P\bar{4}3m$), Γ in SG No. 216 ($F\bar{4}3m$) and Γ, H in SG No. 217 ($I\bar{4}3m$). In contrast, for nonsymmorphic achiral SGs, because of the presence of nonsymmorphic operations (glide mirrors or screw rotations) that complicate the algebra, 4D corepresentations are more widely supported at TRIMs. And notably, the TRIM R in SG No. 218 ($P\bar{4}3n$) and the TRIM H in SG No. 220 ($I\bar{4}3d$) further allows 8D corepresentations, which is consistent with the findings of Wieder et al. in [24] and Bradlyn et al. in [14].

Next, we present some realistic material examples to verify the results in Supplementary Table 5 and show how KNLs emerge out from these TRIMs when higher-dimensional corepresentations are hosted near Fermi level. In Supplementary Fig. 6, we plotted the band structure of HgSe (SG No. 216, $F\bar{4}3m$, T_d), half-Heusler material YPtBi (SG No. 216, $F\bar{4}3m$, T_d), CsPbF3 (SG No. 161, $R3c$, C_{3v}), La_4Bi_3 (SG No. 220, $I\bar{4}3d$, T_d). The energy bands at TRIMs describing by 4D corepresentations are circled in Supplementary Fig. (6**a**, **b**, **f**) and describing by 8D corepresentations are circled in Supplementary Fig. 6**h**. It can be seen that the appearance of higher corepresentations for these space groups are consistent with Supplementary Table 5: The TRIM Γ for symmorphic SG No. 216 ($F\bar{4}3m$), the TRIM Z for nonsymmorphic SG No. 161 ($R3c$) can host 4D corepresentations, while the TRIM H for nonsymmorphic SG No. 220 ($I\bar{4}3d$) can host 8D corepresentations. On the other hand, the KNLs in these band structures are highlighted as blue color. For an achiral noncentrosymmetric space groups, the appearance of high corepresentations at a TRIM can enforce several KNLs touch together at this TRIM (see Supplementary Fig. 6). It can be found that there always are KNLs emerging out from these achiral TRIMs. And it is worth noting that this is also true for the cases when energy bands on TRIMs are captured by higher-dimensional corepresentations.

[1] Kresse, G. & Furthmuller, J. Efficiency of ab-initio total energy calculations for metals and semiconductors using a plane-wave basis set. *Comput. Mater. Sci.* **6**, 15–50 (1996).

[2] Blöchl, P. E. Projector augmented-wave method. *Phys. Rev. B* **50**, 17953–17979 (1994).

[3] Perdew, J. P. & Burke, K. Ernzerhof, M. Generalized gradient approximation made simple. *Phys. Rev. Lett.* **77**, 3865–3868 (1996).

[4] Langreth, D. C. & Mehl, M. J. Beyond the local-density approximation in calculations of ground-state electronic properties. *Phys. Rev. B* **28**, 1809–1834 (1983).

[5] Hohenberg, M. J. & Kohn, W. Inhomogeneous electron gas. *Phys. Rev.* **136**, B864–B871 (1964).

[6] Jain, A., Ong, S.P. et al. The Materials Project: A materials genome approach to accelerating materials innovation. *APL Materials* **1**(1), 011002 (2013).

[7] Hinuma, Y., Pizzi, G. Y. et al. Band structure diagram paths based on crystallography, *Comp. Mat. Sci.* **128**, 140 (2017).

[8] NIST Inorganic Crystal Structure Database, NIST Standard Reference Database Number 3, National Institute of Standards and Technology, Gaithersburg MD, 20899, DOI: https://doi.org/10.18434/M32147.

[9] Autes G., Wu Q., Mounet N., Yazyev O. V., TopoMat: a database of high-throughput first-principles calculations of topological materials, Materials Cloud Archive 2019.0019/v2 (2019), doi: 10.24435/materialscloud:2019.0019/v2.

[10] Marzari, N., & Vanderbilt, D. Maximally localized generalized Wannier functions for composite energy bands, *Phys. Rev. B* **56**, 12847 (1997).

[11] Mostofi, A. A., Yates, J. R. et al. wannier90: A Tool for Obtaining Maximally-Localised Wannier Functions, *Comput. Phys. Commun.* **178**, 685 (2008).

[12] Wu, Q., Zhang, S. et al. WannierTools: An open-source software package for novel topological materials. *Computer Physics Communications* **224**, 405-416 (2018).

[13] Bradley, C. J. & Cracknell, A. P. *The Mathematical Theory of Symmetry in Solids* (Oxford University Press, 2009).

[14] Bradlyn, B. et al. Beyond Dirac and Weyl fermions: unconventional quasiparticles in conventional crystals. *Science* **353**, 5037 (2016).

[15] Zhang, J. et al. Topological band crossings in hexagonal materials. *Phys. Rev. Materials* **2**, 074201 (2018).

[16] Chan, Y.-H. et al. Symmetry-enforced band crossings in trigonal materials: Accordion states and Weyl nodal lines, arXiv:1908.00901.

[17] Elcoro, L. et al. Double crystallographic groups and their representations on the Bilbao Crystallographic Server. *Journal of Applied Crystallography* **50**, 1457 (2017).

[18] Xu, Y. et al. High-throughput calculations of magnetic topological materials. *Nature* **586**, 702–707 (2020).

[19] Elcoro, L. et al. Magnetic topological quantum chemistry. *arXiv*:2010.00598 (2020).

[20] Chang, G. et al. Topological quantum properties of chiral crystals. *Nat. Mater.* **17**, 978-985 (2018).

[21] Bahramy, M. S., Yang, B. J., Arita, R. and Nagaosa, N. Emergence of non-centrosymmetric topological insulating phase in BiTeI under pressure. *Nature Commun.* **3**, 679 (2012).

[22] de Juan, F. et al. Quantized circular photogalvanic efect in Weyl semimetals. *Nat. Commun.* **8**, 15995 (2017).

[23] Sipe, J. E. and Shkrebtii, A. I. Second-order optical response in semiconductors. *Phys. Rev. B* **61**, 5337–5352 (2000).

[24] Wieder, B. J., Kim, Y., Rappe, A. M. & Kane, C. L. Double Dirac semimetals in three dimensions. *Phys. Rev. Lett.* **116**, 186402 (2016).
